# Supplementary material for: Associations between Child Snack and Beverage Consumption, Severe Dental Caries, and Malnutrition in Nepal
Source: Int J Environ Res Public Health. 2020 Oct 28;17(21):7911. doi: 10.3390/ijerph17217911 (PMC7672540; doi:10.3390/ijerph17217911)
Supplement: Supplementary file 1 [file ijerph-17-07911-s001.zip › ijerph-940163-supplementary.docx]

Supplementary materials:

S1 – Mother Interview Form

A ID Number: (IS-01- _) Parent ID:

Child ID:

B Mother's Name:

C If the mother didn't do the interview, who did? Name and Relation to child:

D. Have you had any prior contact with Jevaia Oral Health Care? (Y: clinic/seminar/other) or N) Q1 Mother's Age

Q2 Mother's Years of School Completed Q3 How many children does the mother have?

Q4 a. How many children are younger than 7 years old?

m 0 (1) m 1 (2) m 2 (3) m 3 (4) m 4 (5) m 5 (6) m 6 (7) m 7 (8)

Q4 b. Write the children's name and age.

Name (1) Age (2) Child 1 (1) Child 2 (2) Child 3 (3) Child 4 (4) Additional Child? (5) Q5 How many people live in your house?

Q6 How long does it take you to walk to a store where you can buy snack food (e.g., chips, candy, soda)? Less than 5 minutes (1) 6 to 20 minutes (2) 21 to 40 minutes (3) 41 to 60 minutes

(4) More than 60 minutes (5)

Q7 When do you go to the doctor? Only when I am sick (1) For check-ups (2) Both (3) Truthfully, I never go to the doctor (4) Other (5) _

Q8 When do you go to the dentist?

Only when I have problems with my teeth/molars (1) For check-ups (2) Both (3) Truthfully, I never go to the dentist (4) Other (5)

Q9 a. How often do you drink milk? Never (1) Every 2-4 weeks (2) Every week (3) 2-3 times per week (4) Once a day (5) 2-3 times per day (6)

Q9 b. How often do you drink tea with sugar? Never (1) Every 2-4 weeks (2) Every week (3) 2-3 times per week (4) Once a day (5) 2-3 times per day (6)

Q9 c How often do you drink soda? Never (1) Every 2-4 weeks (2) Every week (3) 2-3 times per week (4) Once a day (5) 2-3 times per day (6)

Q10 How often do you eat chips, biscuits, etc.? Never (1) Every 2-4 weeks (2) Every week (3) 2- 3 times per week (4) Once a day (5) 2-3 times per day (6)

Q11 How often do you eat sweets, candy, chocolate etc.? Never (1) Every 2-4 weeks (2) Every week (3) 2-3 times per week (4) Once a day (5) 2-3 times per day (6)

Q12 How much money do you spend on the children's snack foods per week? I don't buy snack food (1) less than 5 Rupees/week/child (less than 1 rs/day) (2) 5-15 Rupees/week/child (1-2

rs/day) (3) 16-30 Rupees/week/child (between 3-4 rs/day) (4) 31-50 Rupees/week/child (between

5-7 rs/day) (5) 51-70 Rupees/week/child (between 8-10 rs/day) (6) more than 70 Rupees/week/child

(>10 rs/day) (7)

Q13 In your life, how many of your teeth or molars have fallen out or been extracted? (not including baby teeth)

Q14 Right now in your home, do you have your own toothbrush? Yes (1) No (2) Q15 Did you brush your teeth yesterday? Yes (1) No (2)

Q16 How long ago was the last time you went to the dentist? (months) (Never been = 00)

Q17 Why did you go to the dentist the last time? Pain in the tooth/molar (1) Bleeding Gums (2) Decayed tooth/molar (3) Filling of tooth/molar (4) Check-up (5) Because I went with my child

(6) Other (7)

Q18 In the past 3 months, have you had any problem with your teeth, molars, gums, or mouth? (mark all that apply.) Pain or sensitivity (1) Decayed or loose teeth/molars (2) Bleeding gums

(3) Other (4)

Q19 Do you think that oral and dental health can cause problems during pregnancy? Yes (1) No

(2) I don't know (3)

Q20 Do you think that dental treatment causes any of these? Blindness (1) Hearing problems (2) Both (3) I don’t know (4)

Q21 Did you ever experience changes in your oral health when you were pregnant or breastfeeding? Yes (1) No (2) I do not know (3)

Q22 Do you think that caries cause problems for children? Yes (1) No (2) I don't know (3)

Q23 What do you think—what causes caries in baby teeth? (multiple answers OK) I don't know

(1) Sweets (2) Gum (3) Juice/Soda (4) Not brushing your teeth (5) Baby bottle (6) Other (7)

Q24 How do they affect children? I don't know (1) Pain (2) They can't eat (3) They can't sleep

(4) Decayed teeth/molars (5) It harms their health (6) Other (7)

S2 – Child Interview Forms

Q1 Child ID Number Q2: Parent Id No:

Q3 Interviewer (health worker conducting the interview's name) Q4 Child's Full Name (First, Middle, Last):

Q5 Child - Date of Birth Month (1) Day (2) Year (3) Q6 Sex- Male (1) Female (2)

Q8 Did you receive prenatal care when you were pregnant with this child? Yes (1) No (2) Q9 If yes, how many prenatal visits?

Q10 Are the child's vaccinations up to date? Yes (1) No (2) I don't know (3)

Q11 Did you breastfeed this baby? Yes (1) No (2)

Answer Q12 if answered yes to Did you breastfeed this baby?

Q12 If breastfed, to what age? (in months)

Q13 Are you currently still breastfeeding this baby? Yes (1) No (2) Q14 Did you give this child a baby bottle? Yes (1) No (2)

Answer Q15a-b if answered yes to Did you give this child a baby bottle?

Q15a If you gave the baby bottle, to what age?

Q15b Is the baby currently using a baby bottle? Yes (1) No (2)

Q16 How often does he/she fall asleep with the baby bottle in his/her mouth? Never (1) Occasionally (once a week) (2) Frequently (two to three times a week) (3) Almost Always (more than three times a week) (4)

Q17 What does the baby drink in the baby bottle? (mark all that apply) Water (1) Milk (2) Formula (powder) (3) Lemon water (4) Juice (5) Coffee (6) Soda (7) Sugar water (8) Tea (with

sugar) (9) Other (10)

Q18 Do you do any of the following? (Multiple answers OK) Share food with your child (1) Chew food for you child (2) Eat from the same plate (3)

Q19 How often does this child consume the following things? Milk: Never (1) Every 2-3 weeks

(2) Once a week (3) 2-3 Times a Week (4) Daily (5) 2-3 times a day (6)

Q20 How often does this child consume the following things? Soda: Never (1) Every 2-3 weeks

(2) Once a week (3) 2-3 Times a Week (4) Daily (5) 2-3 times a day (6)

Q21 How often does this child consume the following things? Tea with sugar: Never (1) Every 2- 3 weeks (2) Once a week (3) 2-3 Times a Week (4) Daily (5) 2-3 times a day (6)

Q22 How often does this child consume the following things? Sweets, Candy, Chocolate: Never

(1) Every 2-3 weeks (2) Once a week (3) 2-3 Times a Week (4) Daily (5) 2-3 times a day (6)

Q23 How often does this child consume the following things? Chips, biscuits, etc.: Never (1) Every 2-3 weeks (2) Once a week (3) 2-3 Times a Week (4) Daily (5) 2-3 times a day (6)

Q24 When your child cries during day or the night, what do you do to calm him/her? Comfort him/her (1) Hit him/her (2) Give him/her medicine (3) Give him/her sweets (4) Give him/her the breast (5) Give him/her the baby bottle (6) Other (7)

Q25 How much money do you give to your child per day? 1-5 rupees (1) 6-10 rupees (2) 11-15 rupees (3) 16-20 rupees (4) more than 20 rupees (5) I don't give my child money (6)

Q26 What do you do to take care of your child's teeth? Brush them (1) Don't give them candy (2) Nothing (3) Other (4)

Q27 Right now in your home, does your child have his/her own toothbrush? Yes (1) No (2) Q28 Did you child brush his/her teeth yesterday? Yes (1) No (2) I don’t know (3)

Q29 Do you help your child brush his/her teeth? Never (1) Occasionally (once a week) (2) Frequently (two to three times a week) (3) Almost Always (more than three times a week) (4)

Q30 Has your child been to the dentist? Yes (1) No (2)

Answer Q31 if answered yes to Has your child been to the dentist?

Q31 If he/she has been to the dentist, how many times?

Q32 If he/she has been to the dentist, why did he/she go? For a check-up (1) Caries (2) Pain (3) Child has never been to the dentist (4) Other (5) _

**S3 - Odontogram for dental examination**:


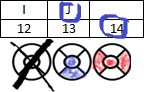


***Legend***

Circle the tooth for number of teeth present. Place an “X” if tooth is missing.

Filling, color the tooth surface: Blue or stripe Decay, color the tooth surface: Red or solid

| PRIMARY/ BABY TEETH | | | | | | | | | | | | | | | |
| --- | --- | --- | --- | --- | --- | --- | --- | --- | --- | --- | --- | --- | --- | --- | --- |
|  |  |  | A | B | C | D | E | F | G | H | I | J |  |  |  |
| 1 | 2 | 3 | 4 | 5 | 6 | 7 | 8 | 9 | 10 | 11 | 12 | 13 | 14 | 15 | 16 |
| PERMANENT/ADULT TEETH | | | | | | | | | | | | | | | |

Maxillary (top teeth) CHEEK SIDE


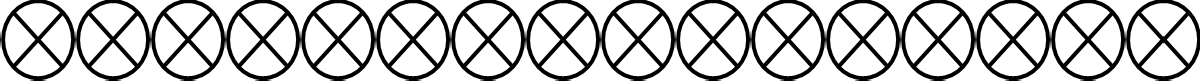

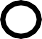

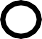

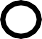

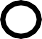

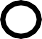

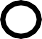

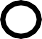

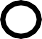

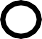

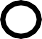

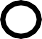

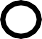

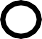

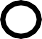

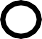

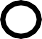


TONGUE SIDE

| PRIMARY/ BABY TEETH | | | | | | | | | | | | | | | |
| --- | --- | --- | --- | --- | --- | --- | --- | --- | --- | --- | --- | --- | --- | --- | --- |
|  |  |  | T | S | R | Q | P | O | N | M | L | K |  |  |  |
| 32 | 31 | 30 | 29 | 28 | 27 | 26 | 25 | 24 | 23 | 22 | 21 | 20 | 19 | 18 | 17 |
| PERMANENT/ADULT TEETH | | | | | | | | | | | | | | | |

Mandibular (bottom teeth) TONGUE SIDE


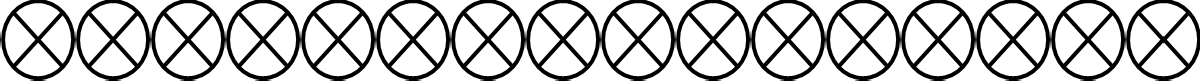

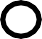

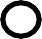

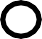

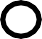

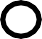

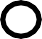

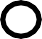

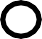

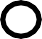

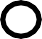

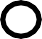

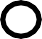

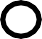

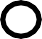

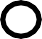

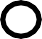


CHEEK SIDE
